# Oral nicotinamide riboside raises NAD+ and lowers biomarkers of neurodegenerative pathology in plasma extracellular vesicles enriched for neuronal origin

**DOI:** 10.1111/acel.13754

**Published:** 2022-12-14

**Authors:** Michael Vreones, Maja Mustapic, Ruin Moaddel, Krishna A. Pucha, Jacqueline Lovett, Douglas R. Seals, Dimitrios Kapogiannis, Christopher R. Martens

**Affiliations:** ^1^ Human Neuroscience Section National Institute on Aging Baltimore Maryland USA; ^2^ Department of Integrative Physiology University of Colorado Boulder Boulder Colorado USA; ^3^ Department of Kinesiology & Applied Physiology University of Delaware Newark Delaware USA

**Keywords:** extracellular vesicles, NAD^+^, NADH, neurodegenerative disease

## Abstract

Declining nicotinamide adenine dinucleotide (NAD^+^) concentration in the brain during aging contributes to metabolic and cellular dysfunction and is implicated in the pathogenesis of aging‐associated neurological disorders. Experimental therapies aimed at boosting brain NAD^+^ levels normalize several neurodegenerative phenotypes in animal models, motivating their clinical translation. Dietary intake of NAD^+^ precursors, such as nicotinamide riboside (NR), is a safe and effective avenue for augmenting NAD^+^ levels in peripheral tissues in humans, yet evidence supporting their ability to raise NAD^+^ levels in the brain or engage neurodegenerative disease pathways is lacking. Here, we studied biomarkers in plasma extracellular vesicles enriched for neuronal origin (NEVs) from 22 healthy older adults who participated in a randomized, placebo‐controlled crossover trial (NCT02921659) of oral NR supplementation (500 mg, 2x /day, 6 weeks). We demonstrate that oral NR supplementation increases NAD^+^ levels in NEVs and decreases NEV levels of Aβ42, pJNK, and pERK1/2 (kinases involved in insulin resistance and neuroinflammatory pathways). In addition, changes in NAD(H) correlated with changes in canonical insulin–Akt signaling proteins and changes in pERK1/2 and pJNK. These findings support the ability of orally administered NR to augment neuronal NAD^+^ levels and modify biomarkers related to neurodegenerative pathology in humans. Furthermore, NEVs offer a new blood‐based window into monitoring the physiologic response of NR in the brain.

## Graphical abstract

Oral nicotinamide riboside (NR) supplementation increased nicotinamide adenine dinucleotide (NAD+) in extracellular vesicles enriched for neuronal origin (NEVs) and decreased biomarkers of neurodegenerative disease and insulin signaling in late middle‐aged and older adults.

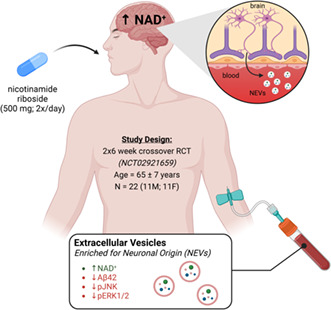

Nicotinamide adenine dinucleotide (oxidized form, NAD^+^) is an essential metabolite involved in a wide range of longevity‐related cellular processes. Classically, NAD^+^ functions as a critical coenzyme in cellular metabolism by accepting and shuttling the reducing power generated through metabolic redox reactions to the mitochondria, where NADH is used to generate ATP through oxidative phosphorylation. More recently, NAD^+^ has emerged as a critical regulator of NAD^+^‐dependent enzymes that mediate cellular signaling pathways related to metabolism, epigenetic gene regulation, and mitochondrial function, among several other survival‐associated mechanisms (Covarrubias et al., 2021). Accordingly, declining NAD^+^ concentration in several tissues has emerged as a common pathological factor in various aging‐associated diseases (Katsyuba et al., 2020; McReynolds et al., 2020).

The brain is particularly vulnerable to alterations in NAD^+^ content during aging due to the high energetic demand of neurons, and brain‐specific decline in NAD+ concentration during aging has been reported in several species, including humans (Mouchiroud et al., 2013; Zhu et al., 2015). Restoring brain NAD^+^ concentration using the dietary NAD^+^ precursor nicotinamide riboside (NR) has shown strong efficacy in animal models and has improved key features of neurodegenerative disorders, including amyloid‐beta (Aβ) and tau pathologies (hallmarks of Alzheimer's disease (AD)), neuroinflammation, and mitochondrial dysfunction (Hou et al., 2021; Schöndorf et al., 2018; Xie et al., 2019). Interestingly, brain‐specific insulin resistance is thought to be a major contributor to these neurodegenerative cascades (Mullins et al., 2017). NAD^+^ supplementation can protect peripheral tissues against insulin resistance (Stromsdorfer et al., 2016; Trammell et al., 2016; Yoshino et al., 2021), although similar effects have yet to be demonstrated for the brain. Overall, augmenting brain‐based NAD^+^ metabolism has emerged as a promising strategy for the treatment of neurological disorders (Brakedal et al., 2022; Verdin, 2015).

While the preclinical evidence for the use of NR and other NAD^+^ precursors in neurological disorders is promising, evidence supporting their ability to augment brain NAD^+^ concentration or alter disease biomarkers in humans remains scarce. Furthermore, their capacity to engage insulin signaling pathways in the brain has yet to be explored. Here, we leveraged plasma extracellular vesicles (EVs) enriched for neuronal origin (NEVs) (Mustapic et al., 2017) to assess changes in neuronal NAD^+^ and NADH concentration, in response to oral supplementation with NR in healthy middle‐aged and older adults. We analyzed plasma samples from 22 healthy older adults (11 M/11F; 65 ± 7 years old) who completed a previously published double‐blind, placebo‐controlled, crossover clinical trial of oral NR (500 mg, 2x/day; 6 weeks), which reported an increase in blood‐cellular NAD^+^ concentration and improved cardiovascular parameters following NR administration (Martens et al., 2018). Additional details of the clinical trial are provided in the supplemental methods and on clinicaltrials.gov (NCT02921659). We also measured NEV levels of AD biomarkers Aβ42, p‐Tau‐181, and total Tau due to the strong preclinical evidence demonstrating engagement of these disease‐specific pathways by NAD^+^ boosting molecules, such as NR and nicotinamide mononucleotide (NMN). Finally, in an exploratory fashion, we measured phosphoproteins involved in insulin signaling (pSer312‐IRS‐1, pS473‐Akt, pS9‐GSK3‐B, pThr421/Ser424‐p70S6K, pJNK, pERK1/2, and pp38), motivated by the ability of NAD^+^ precursors to modulate peripheral insulin resistance (Trammell et al., 2016; Yoshino et al., 2011). We hypothesized that NR is capable of increasing NAD^+^ concentration in NEVs, which we consider a surrogate for a commensurate change in their parental neurons. We also hypothesized that changes in NEV NAD^+^ concentration following NR supplementation would be related to changes in biomarkers of AD pathology and insulin signaling.

NEVs were enriched using an immunoaffinity capture method targeting a sub‐population of plasma EVs that express the neuronal marker L1CAM (described in detail in Mustapic et al. (2017)). To evaluate the quality of our NEV preparations, we assessed canonical‐positive and canonical‐negative EV markers in NEVs, total EVs, and EV‐depleted plasma via immunoblotting according to established guidelines (Thery et al., 2018). Our NEV preparations abundantly express the EV markers ALIX and CD9 and are relatively depleted of non‐EV associated cellular debris and plasma lipoproteins, as evidenced by the decreased expression of Golgi marker GM130 and APOA1, respectively (Supporting information Figure S1a). Nanoparticle tracking analysis (NTA) revealed a size distribution consistent with existing EV literature, with the majority of particles being between 150 and 250 nm. The size distribution and average concentration of NEVs were similar between treatment groups (Supporting information Figure S1b). NEV concentration was used to normalize protein biomarker values to account for differential NEV recovery, as performed previously (Eitan et al., 2017; Eren et al., 2020). Lastly, we demonstrate NEV enrichment for neuronal markers L1CAM and β‐III‐tubulin, with 1.6 and 3.7 fold differences between NEVs and total circulating EVs, respectively (Supporting information Figure S1c).

We measured NAD^+^ and NADH in NEV isolates using a previously developed LC–MS method (Demarest et al., 2019). Interestingly, while previously reported to be below quantitation in plasma, our enriched NEVs had NADH levels above quantitation. NAD^+^, however, could be detected but not quantified; therefore, a more sensitive assay was sought (luciferase‐based plate assay, Promega) and quantitative NAD^+^ measurements were obtained for 10 of the 22 subjects (five males and five females). As shown in Figure 1a, NAD^+^ levels in NEVs were significantly greater after treatment with NR when compared to placebo (paired *t*test, *p* = 0.0092), while NEV levels of NADH showed no differences (paired *t*test, *p* = 0.215). Consistent with a previous report examining the effects of NR on brain‐specific changes in NAD^+^ levels (Brakedal et al., 2022), 9/10 subjects with successfully quantified levels showed increased NEV NAD^+^ following NR. To address the possibility of heterogeneous treatment effects, we chose to include a responder subgroup (*n* = 9 participants with documented NAD^+^ increases; five males and four females) in all subsequent analyses.

**FIGURE 1:**
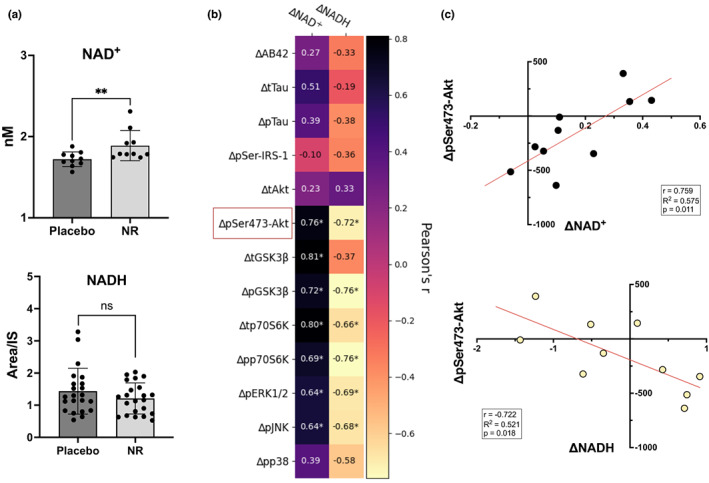
NAD^+^ and NADH concentrations in NEVs and change–change correlations with insulin signaling proteins. (a) Concentration of NAD+ after 6 weeks of oral nicotinamide riboside (NR) supplementation was significantly higher in NEVs when compared to placebo (*n* = 10, *p* = 0.0092, paired *t*test), while NADH remained relatively unchanged (*n* = 22, *p* = 0.215, paired *t*test). Bars represent means, and error bars represent SDs. (b) Changes in NAD^+^ concentration were positively correlated with changes in pSer473‐Akt, tGSK3β, pGSK3β, tp70S6K, pp70S6K, pERK1/2, and pJNK. Changes in NADH were negatively correlated with changes in pSer473‐Akt, pGSK3β, tp70S6K, pp70S6K, pERK1/2, and pJNK. Numbers inside cells represent Pearson's correlation coefficients; * denotes significance <0.05. Red square depicts analyte selected for visualization in (c). (c) Change to change correlation plots between NAD^+^ or NADH and NEV protein biomarkers (*n* = 10).

The concentration of Aβ42, p‐Tau‐181, and total Tau in NEVs did not change following NR relative to placebo when analyzing all participants; however, we observed a significant decrease in Aβ42 relative to placebo (Figure 2a, *p* = 0.015) in the responder subgroup. In the entire cohort, we observed no significant changes in pSer‐IRS‐1, pAkt, pGSK3β, or pp70S6K or their total concentrations following NR compared with placebo (Figure 2b). Moreover, NEV levels of the mitogen‐activated protein kinases pJNK and pp38 were unchanged following NR compared with placebo, while pERK1/2 showed a marginal decrease (Figure 2c, *p* = 0.066). In the responder subgroup, we observed a significant decrease in pJNK (*p* = 0.036) and pERK1/2 (*p* = 0.038) following NR compared with placebo.

**FIGURE 2:**
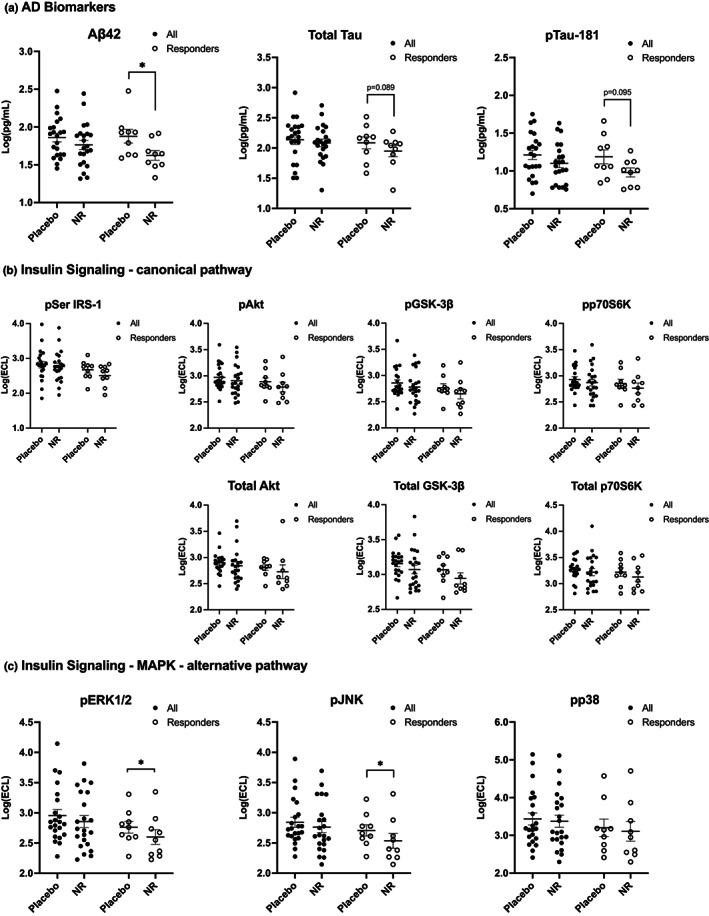
NEV biomarkers in response to oral nicotinamide riboside supplementation. (a) Alzheimer's disease biomarkers. (b) Canonical insulin/Akt signaling mediators. (c) Alternative MAPK insulin signaling biomarkers. Two‐tailed paired *t*test was used for all analyses; horizontal bars depict mean; error bars depict SEM. * indicates significance >0.05.

We hypothesized that the effects of NR on NEV protein biomarkers would be, at least in part, linked to changes in NAD^+^ levels. Therefore, we explored correlations between changes in NEV NAD^+^ and NADH and changes in NEV proteins. Changes in pAkt, tGSK3β, pGSK3β, tp70S6K, pp70S6K, pERK1/2, and pJNK were significantly positively correlated with changes in NAD^+^ and negatively correlated with changes in NADH (except for tGSK3β; Figure 1b). To facilitate the understanding of the directionality of these change–change correlations, we show representative plots for ΔpSer473‐Akt vs. ΔNAD^+^ and ΔNADH, Figure 1c.

In conclusion, we report the first study examining NEV biomarkers in response to oral NR supplementation. Our primary analysis of NAD^+^ and NADH in NEVs suggests an increase in neuronal NAD^+^ concentration in response to oral NR supplementation. To gain further mechanistic insights, we performed exploratory analyses, which revealed decreases in NEV concentrations of Aβ42, pJNK, and pERK1/2 following NR compared with placebo, specifically among NAD^+^ responders. We hypothesized that NEV‐phosphorylated levels of MAP kinases JNK, ERK1/2, and p38, an alternative branch of the insulin cascade, would be boosted by NR supplementation through enhanced overall insulin signaling in neurons. Instead, our findings suggest that NR may decrease JNK and ERK1/2 phosphorylation. Interestingly, JNK is highly phosphorylated and co‐localizes with Aβ in human post‐mortem AD brain (Zhu et al., 2001), and NAD^+^ augmentation attenuates both Aβ accumulation and JNK activation in transgenic AD mice (Yao et al., 2017). Hyperactivation of ERK1/2 has also been linked to AD (Ferrer et al., 2006; Pei et al., 2002). Therefore, NR might hold therapeutic value in the context of AD by concomitantly reducing Aβ burden and the activity of JNK and ERK1/2.

A mechanism by which NR may alter cellular functions is by increasing NAD^+^. To further explore whether changes in NAD+ are mechanistically linked with changes in other NEV biomarkers, we examined change–change correlations between NEV biomarkers following NR relative to placebo. The strong positive correlations between changes in NAD^+^ and changes in Akt signaling phosphoproteins, and pERK1/2 and pJNK in NEVs suggest engagement of the insulin signaling cascade (downstream of IRS‐1) in their parent neurons by NR (Figure 2c). Interestingly, change–change correlations between NADH or NAD^+^, and NEV biomarkers were similar in strength, yet opposite in direction, perhaps, reflecting the redox coupling of these molecules. On the contrary, changes in Aβ42 did not correlate with changes in NAD^+^, suggesting that NR may have pleiotropic effects and that the Aβ42 decrease was mechanistically unrelated. Multiple studies have demonstrated reductions in amyloid pathology following NR treatment in aging and genetically modified mice. NR treatment increases expression of PGC1‐α in the brain, which downregulates the production of Aβ42 by facilitating the degradation of BACE1, which catalyzes the first cleavage in the amyloidogenic processing of APP (Gong et al., 2013). The resulting reduced Aβ42 production may be reflected on reduced NEV Aβ42 levels with NR treatment compared with placebo. Furthermore, NR treatment enhances mitophagy and mitochondrial function, which, in turn, promotes the clearance of extracellular amyloid via enhanced phagocytosis by microglia (Fang et al., 2014, 2016, 2019). Thus, NR treatment may engage multiple mechanisms modulating Aβ42 production and clearance even independent of its effects on NAD^+^.

Our results provide insights into neuronal effects of NR in living humans and highlight the utility of circulating NEVs as a means of demonstrating target engagement in clinical trials. We also show that the utility of NEVs as a source of biomarkers may be expanded beyond proteins to include small molecules, such as NAD^+^ and NADH. While we believe our study contains strengths, such as the methodologically robust design of the clinical trial and the breadth of biomarkers measured, we report our findings with caution due to the limited number of subjects and our inability to quantify NAD^+^ for the entire cohort.

## AUTHOR CONTRIBUTIONS

C.R.M. and D.R.S. conducted the original clinical trial and provided all the plasma samples for this study. C.R.M. and D.K. conceived the idea for this study and developed the overall study design. M.V., M.M., R.M., K.P., and J.L. isolated the extracellular vesicles from plasma samples and developed and ran all assays to measure NAD^+^/NADH and biomarkers of neurodegenerative disease. M.V., D.K., and C.M. analyzed the data and drafted the manuscript. All authors edited and approved the final manuscript.

## CONFLICT OF INTEREST

The authors declare no competing interests.

## Supporting information


Supinfo S1

**Figure S1** Characterization of neuronal origin‐derived extracellular vesicles (NEVs) isolated from plasma via L1CAM immunoprecipitation. (a) Representative Western blot images and quantification of canonical‐positive (ALIX and CD9) and canonical‐negative (GM130 and APOA1) EV markers in NEVs, total EVs, and EV‐depleted plasma (ordinary one‐way ANOVA, *n* = 3). (b) Treatment group analysis of size distributions, mean size, mean concentration, and mode size of NEV isolates as determined by nanoparticle tracking analysis. Vertical lines depict SD. (c) Western blot images and quantification of neuronal marker β‐III‐tubulin, immunoprecipitation target L1CAM, and EV marker CD9 in NEVs, total EVs, and EV‐depleted plasma. Human brain lysate was used as a positive control for neuronal proteins (unpaired *t*test, *n* = 3). Images analyzed using FIJI/Image J. CD9 used as loading control; error bars depict SD; *indicates significance <0.05; **indicates significance <0.005.
**FIGURE S2** Calibration curve used to interpolate NAD+ concentrations from Promega NAD+/NADH luciferase assay. NAD+ Standards were created at concentrations of 10 nM, 5 nM, 2.5 nM, 1.25 nM, and 0.625 nM.
**FIGURE S3** NADH levels in L1CAM+ EVs in responders only. Even when analyzing responders (defined as subjects with documented NAD+ increases in L1CAM+ EVs) only (*n* = 9), levels of NADH in plasma extracellular vesicles enriched for neuronal origin remain relatively unchanged after oral NR supplementation when compared to the placebo condition (two‐tailed paired *t*test, *p* = 0.44).

## Data Availability

The data that support the findings of this study are available from the corresponding author upon reasonable request.
