# Supplementary material for: Oral nicotinamide riboside raises NAD+ and lowers biomarkers of neurodegenerative pathology in plasma extracellular vesicles enriched for neuronal origin
Source: Aging Cell. 2022 Dec 14;22(1):e13754. doi: 10.1111/acel.13754 (PMC9835564; doi:10.1111/acel.13754)
Supplement: Supplementary file 1 — Supinfo S1 [file ACEL-22-e13754-s002.docx]

**Methods and Materials**

**Clinical Trial**

De-identified plasma samples were obtained from a previously conducted clinical trial of NR supplementation in 24 healthy late middle-aged and older adults (NCT02921659); however, extra plasma samples were only available for 22 participants (11 male, 11 female; 65 ± 7 years old). Detailed information on the study design, baseline subject characteristics, and primary outcomes have been reported elsewhere (Martens et al., 2018). The study design consisted of a double-blind, placebo-controlled crossover study in which subjects consumed NR (NIAGEN; 500 mg, 2x/day; ChromaDex, Inc.) and placebo for 6 weeks each in a randomized order. All comparisons were made between the end of the placebo phase and the end of the NR phase. Subjects refrained from taking study capsules within 12-hours of each blood draw to avoid any acute effects of NR on blood-based biomarkers.

**Isolation of neuron-derived extracellular vesicles from blood plasma**

Plasma samples were thawed on ice and extracellular vesicles enriched for neuronal origin (NEVs) were immediately enriched using a well characterized methodology (Mustapic et al., 2017). Briefly, 0.5 µl plasma aliquots were incubated with 6µl thrombin (System Biosciences, Inc., Mountainview, CA) for 30 min at RT to eliminate coagulant proteins thought to interfere with plasma EV recovery. After addition of 494 µl of 1X Dulbecco’s PBS supplemented with protease and phosphatase inhibitors, samples were centrifuged at 6,000x g for 20 minutes at 4°C. Total EVs were sedimented from resulting supernatants by incubation with 252 µl ExoQuick (System Biosciences) for 1 hr at 4°C, followed by centrifugation at 1,500x g for 20 min at 4°C. The EV depleted supernatant was collected and stored at -80°C for use in Western blot experiments. Total EV pellets were resuspended via overnight rotation in 700 µl ultrapure water supplemented with protease and phosphatase inhibitors. NEVs were enriched from each total EV suspension by incubation with 4 µg of biotinylated anti-human CD171 (neural cell adhesion molecule L1CAM; clone 5G3, eBioscience, San Diego, CA) for 2 hrs at RT, followed by incubation with 25 µl of washed Pierce Streptavidin Plus UltraLink Resin (Thermo Fisher Scientific, Waltham, MA) for 1 hr at RT. Suspensions were centrifuged at 800x g for 10 min at 4°C to pellet EV-antibody-bead complexes and supernatants containing unbound EVs and soluble proteins were removed. Pellets containing NEVs were centrifuged again at 800x g for 10 min at 4°C in the presence of 50 µl ultrapure water to further reduce contamination. After removal of the supernatant, bound NEVs were eluted for 10 min on ice using 200 µl of 0.1M glycine (pH=3). Beads were sedimented by centrifugation at 4,500x g for 5 min at 4°C, and supernatants containing NEVs were immediately neutralized with 30 µl of 1M tris-HCl (pH=8).

10 µL of intact NEVs were separated to measure particle size and concentration using nanoparticle tracking analysis (NTA) (Nanosight NS500; Malvern, Amesbury, UK), while the remaining sample received 50 µl of 3% BSA and 230 µl of Mammalian Protein Extraction Reagent (M-PER; Thermo Fisher Scientific) supplemented with protease and phosphatase inhibitors. 500 µl NEV isolates were lysed for 30 min on ice and freeze thawed twice before storage at -80°C. A variation of this protocol, in which samples received 4µg each of biotinylated CD9 (Cat. #558749, BD Pharmingen, San Diego, CA), CD81 (Cat. #302-030, Ancell, Bayport, MN), and CD63 (Cat. #MAB15361, Abnova, Taiwan), was used to generate the total EV samples used in immunoblot experiments.

**Quantification of NEV Proteins by Immunoblotting and ELISAs**

To confirm the presence of EVs in our NEV preparations, we assessed canonical positive and negative EV markers in NEVs, total EVs, and EV depleted plasma via immunoblotting according to established guidelines (Thery et al., 2018). We also assessed the efficiency of our immunoprecipitation and the neuronal origin of L1CAM+ EVs by probing for L1CAM and beta-III-tubulin, respectively. Protein concentrations were determined for each sample using the Pierce BCA protein assay (Thermo Fisher Scientific) and used to normalize sample loading in each experiment. Protein was resolved by sodium dodecyl sulfate polyacrylamide gel electrophoresis (SDS-PAGE) using precast 4-12% Bis-Tris gels in MOPS SDS running buffer at 200 V for 40 minutes (NuPAGE; Thermo Fisher Scientific) and transferred to polyvinylidene fluoride membranes using the iBlot2 transfer system (Thermo Fisher Scientific). Membranes were blocked for 1 hr at RT using 5% milk in TBS-T and incubated overnight at 4°C with primary antibodies against intravesicular EV marker ALIX (Ca. # ab88388, Abcam, Cambridge, MA), tetraspannin membrane EV marker CD9 (clone H19a, BioLegend, San Diego, CA), negative EV marker GM130 (Cat. #G7295, Sigma Aldrich, Burlington, MA), lipoprotein indicator APOA1 (Ca. # AF3664, R&D Systems, Minneapolis, MN), and neuronal markers L1CAM (Ca. # ab24345, Abcam) and β-III-tubulin (Ca. # ab18207, Abcam). Membranes were washed 3x with TBS-T for 5 min each, followed by incubation with HRP conjugated secondary antibodies (Cat. #7076, Cell Signaling Technology, MA, USA) for 1 hour at RT. Membranes were washed 4x with TBS-T for 10 min each, incubated with ECL substrate (Cat. # RPN2232, Cytiva, Marlborough, MA) for 5 min, and read using the Azure Sapphire Biomolecular Imager (Azure biosystems, Dublin, CA).

Enzyme linked immunosorbent assays (ELISAS) for phospho-p38 (Thr180/Tyr182), phospho-ERK1/2 (Thr202/Tyr204; Thr185/Tyr187), phospho-JNK(Thr183/Tyr185) (Cat. # K15157D), phospho-IRS-1 (Ser312) (Cat. # K150HLD), phospho-Akt (Ser473), phospho-GSK-3β (Ser9), phospho-p70S6K (Thr421/Ser424) (Cat. # K15115D), total Akt, total GSK-3β, total p70S6K (Cat. # K15133D) (Meso Scale Diagnostics, Rockville, MD), Aβ42, total Tau, and phospho-Tau (Thr181) (Cat. # HNABTMAG-68K) (Millipore Sigma, Burlington, MA) were used to quantify human NDEV proteins. NDEV lysates were assayed in duplicate and the average coefficients of variance for each assay were 4.0% (pp38), 8.0% (pERK1/2), 7.9% (pJNK), 20.1% (p-Ser-IRS-1), 10.0% (pAkt), 10.7% (pGSK-3B), 8.4% (pp70S6K), 16.7% (tAkt), 6.5% (tGSK-3B), 4.9% (tp70S6K), 7.2% (Aβ42), 21.0% (tTau), and 7.72% (pTau).

For AB42, total Tau, and phospho-Tau, fluorescent signals were converted to concentrations using a standard curve provided by the manufacturer. The lowest limit of quantification (LLOQ) for each analyte was calculated using the Bio-Plex Manager 6.0 Software (Bio-Rad, Hercules, CA). Four samples were below the LLOQ, but above the limit of detection (LOD; defined as the mean of the blank plus 2.5SD of the blank) with CV values <20%. Therefore, we assigned them the LLOQ value (4 samples total). All other samples were within the linear range of the assay. Protein standards were not provided for assays manufactured by Meso Scale Discovery, therefore, we analyzed the electrochemiluminescent signal using the MESO QuickPlex SQ 120 imager and the Discovery Workbench 4.0 software. Relative particle concentrations for each sample were used to normalize differential EV recovery, as performed previously (Chawla et al., 2019; Eitan et al., 2017). ELISAs were completed blindly without knowledge of the identity of any subject.

**Quantification of NAD+ and NADH in NDEVs**

**NAD+:** NAD+ levels in NEV lysates were assayed using the NAD+/NADH Glo Assay (Cat. # G9071, Promega, Madison, WI), according to the manufacturer’s instructions. A standard curve was constructed using purified NAD+ (Cat. # N0632, Sigma Aldrich, Burlington, MA) to convert bioluminescent signals to concentrations. The average CV for the assay was 6.7% and the signal for all samples fell within the linear range of the curve.

**NADH**: 0.5 mL plasma aliquots were thawed at room temperature (RT) and defibrinated by incubation with 6 µl Thrombin at 30 min at RT. 494 µl of PBS supplemented with protease and phosphatase inhibitors was added, followed by centrifugation at 6,000x g for 20 minutes at 4°C. The supernatant was removed and incubated with 252 µl of Exoquick solution for 1 hour at 4°C, followed by centrifugation at 1,500x for 20 minutes at 4°C. The EV depleted supernatant was removed, and 700 µl of ultrapure water supplemented with protease and phosphatase inhibitors was added to each sample. Total EV pellets were resuspended by overnight rotation at 4°C. The following morning, 4 µg of L1CAM antibody in 3% BSA solution was added to each sample, followed by incubation for 2 hours at 4°C. Samples were then incubated with 25 µl of streptavidin coated beads for 1 hour at 4°C, followed by centrifugation at 800x g for 10 minutes at 4°C. The supernatant was removed, and the beads were spun again at 800x g for 10 minutes with 50 µl of ultrapure water as a decontamination step. The bead-antibody-EV complexes were frozen at -80°C until further processing. The samples were thawed and a 140 µl of 80% methanol was added and 10 µl of internal standard and the samples were processed as previously described and analyzed by LC-MS/MS (Demarest et al., 2019).

**Statistics**

Paired t-tests were used to assess group differences between the Placebo and NR conditions. If data was not normally distributed, then it was log transformed. Pearson correlation coefficients were computed for the relationship between the change in NAD+ or change in NADH vs. the change in insulin signaling proteins.

**References**

Chawla, S., Gulyani, S., Allen, R. P., Earley, C. J., Li, X., Van Zijl, P., & Kapogiannis, D. (2019). Extracellular vesicles reveal abnormalities in neuronal iron metabolism in restless legs syndrome. *Sleep, 42*(7). doi:10.1093/sleep/zsz079

Eitan, E., Tosti, V., Suire, C. N., Cava, E., Berkowitz, S., Bertozzi, B., . . . Fontana, L. (2017). In a randomized trial in prostate cancer patients, dietary protein restriction modifies markers of leptin and insulin signaling in plasma extracellular vesicles. *Aging Cell, 16*(6), 1430-1433. doi:10.1111/acel.12657

Mustapic, M., Eitan, E., Werner, J. K., Jr., Berkowitz, S. T., Lazaropoulos, M. P., Tran, J., . . . Kapogiannis, D. (2017). Plasma Extracellular Vesicles Enriched for Neuronal Origin: A Potential Window into Brain Pathologic Processes. *Front Neurosci, 11*, 278. doi:10.3389/fnins.2017.00278

Thery, C., Witwer, K. W., Aikawa, E., Alcaraz, M. J., Anderson, J. D., Andriantsitohaina, R., . . . Zuba-Surma, E. K. (2018). Minimal information for studies of extracellular vesicles 2018 (MISEV2018): a position statement of the International Society for Extracellular Vesicles and update of the MISEV2014 guidelines. *J Extracell Vesicles, 7*(1), 1535750. doi:10.1080/20013078.2018.1535750

Chawla, S., Gulyani, S., Allen, R. P., Earley, C. J., Li, X., Van Zijl, P., & Kapogiannis, D. (2019). Extracellular vesicles reveal abnormalities in neuronal iron metabolism in restless legs syndrome. *Sleep*, *42*(7). <https://doi.org/10.1093/sleep/zsz079>

Demarest, T. G., Truong, G. T. D., Lovett, J., Mohanty, J. G., Mattison, J. A., Mattson, M. P., Ferrucci, L., Bohr, V. A., & Moaddel, R. (2019). Assessment of NAD+metabolism in human cell cultures, erythrocytes, cerebrospinal fluid and primate skeletal muscle. *Analytical Biochemistry*, *572*, 1-8. <https://doi.org/10.1016/j.ab.2019.02.019>

Eitan, E., Tosti, V., Suire, C. N., Cava, E., Berkowitz, S., Bertozzi, B., Raefsky, S. M., Veronese, N., Spangler, R., Spelta, F., Mustapic, M., Kapogiannis, D., Mattson, M. P., & Fontana, L. (2017). In a randomized trial in prostate cancer patients, dietary protein restriction modifies markers of leptin and insulin signaling in plasma extracellular vesicles. *Aging Cell*, *16*(6), 1430-1433. <https://doi.org/10.1111/acel.12657>

Martens, C. R., Denman, B. A., Mazzo, M. R., Armstrong, M. L., Reisdorph, N., McQueen, M. B., Chonchol, M., & Seals, D. R. (2018). Chronic nicotinamide riboside supplementation is well-tolerated and elevates NAD+ in healthy middle-aged and older adults. *Nature Communications*, *9*(1). <https://doi.org/10.1038/s41467-018-03421-7>

Mustapic, M., Eitan, E., Werner, J. K., Jr., Berkowitz, S. T., Lazaropoulos, M. P., Tran, J., Goetzl, E. J., & Kapogiannis, D. (2017). Plasma Extracellular Vesicles Enriched for Neuronal Origin: A Potential Window into Brain Pathologic Processes. *Front Neurosci*, *11*, 278. <https://doi.org/10.3389/fnins.2017.00278>

Thery, C., Witwer, K. W., Aikawa, E., Alcaraz, M. J., Anderson, J. D., Andriantsitohaina, R., Antoniou, A., Arab, T., Archer, F., Atkin-Smith, G. K., Ayre, D. C., Bach, J. M., Bachurski, D., Baharvand, H., Balaj, L., Baldacchino, S., Bauer, N. N., Baxter, A. A., Bebawy, M., . . . Zuba-Surma, E. K. (2018). Minimal information for studies of extracellular vesicles 2018 (MISEV2018): a position statement of the International Society for Extracellular Vesicles and update of the MISEV2014 guidelines. *J Extracell Vesicles*, *7*(1), 1535750. <https://doi.org/10.1080/20013078.2018.1535750>
