# Supplementary material for: Oral nicotinamide riboside raises NAD+ and lowers biomarkers of neurodegenerative pathology in plasma extracellular vesicles enriched for neuronal origin
Source: Aging Cell. 2022 Dec 14;22(1):e13754. doi: 10.1111/acel.13754 (PMC9835564; doi:10.1111/acel.13754)
Supplement: Supplementary file 2 — Figure S1 Characterization of neuronal origin‐derived extracellular vesicles (NEVs) isolated from plasma via L1CAM immunoprecipitation. (a) Representative Western blot images and quantification of canonical‐positive (ALIX and CD9) and canonical‐negative (GM130 and APOA1) EV markers in NEVs, total EVs, and EV‐depleted plasma (ordinary one‐way ANOVA, n = 3). (b) Treatment group analysis of size distributions, mean size, mean concentration, and mode size of NEV isolates as determined by nanoparticle tracking analysis. Vertical lines depict SD. (c) Western blot images and quantification of neuronal marker β‐III‐tubulin, immunoprecipitation target L1CAM, and EV marker CD9 in NEVs, total EVs, and EV‐depleted plasma. Human brain lysate was used as a positive control for neuronal proteins (unpaired ttest, n = 3). Images analyzed using FIJI/Image J. CD9 used as loading control; error bars depict SD; *indicates significance <0.05; **indicates significance <0.005. FIGURE S2 Calibration curve used to interpolate NAD+ concentrations from Promega NAD+/NADH luciferase assay. NAD+ Standards were created at concentrations of 10 nM, 5 nM, 2.5 nM, 1.25 nM, and 0.625 nM. FIGURE S3 NADH levels in L1CAM+ EVs in responders only. Even when analyzing responders (defined as subjects with documented NAD+ increases in L1CAM+ EVs) only (n = 9), levels of NADH in plasma extracellular vesicles enriched for neuronal origin remain relatively unchanged after oral NR supplementation when compared to the placebo condition (two‐tailed paired ttest, p = 0.44). [file ACEL-22-e13754-s001.docx]

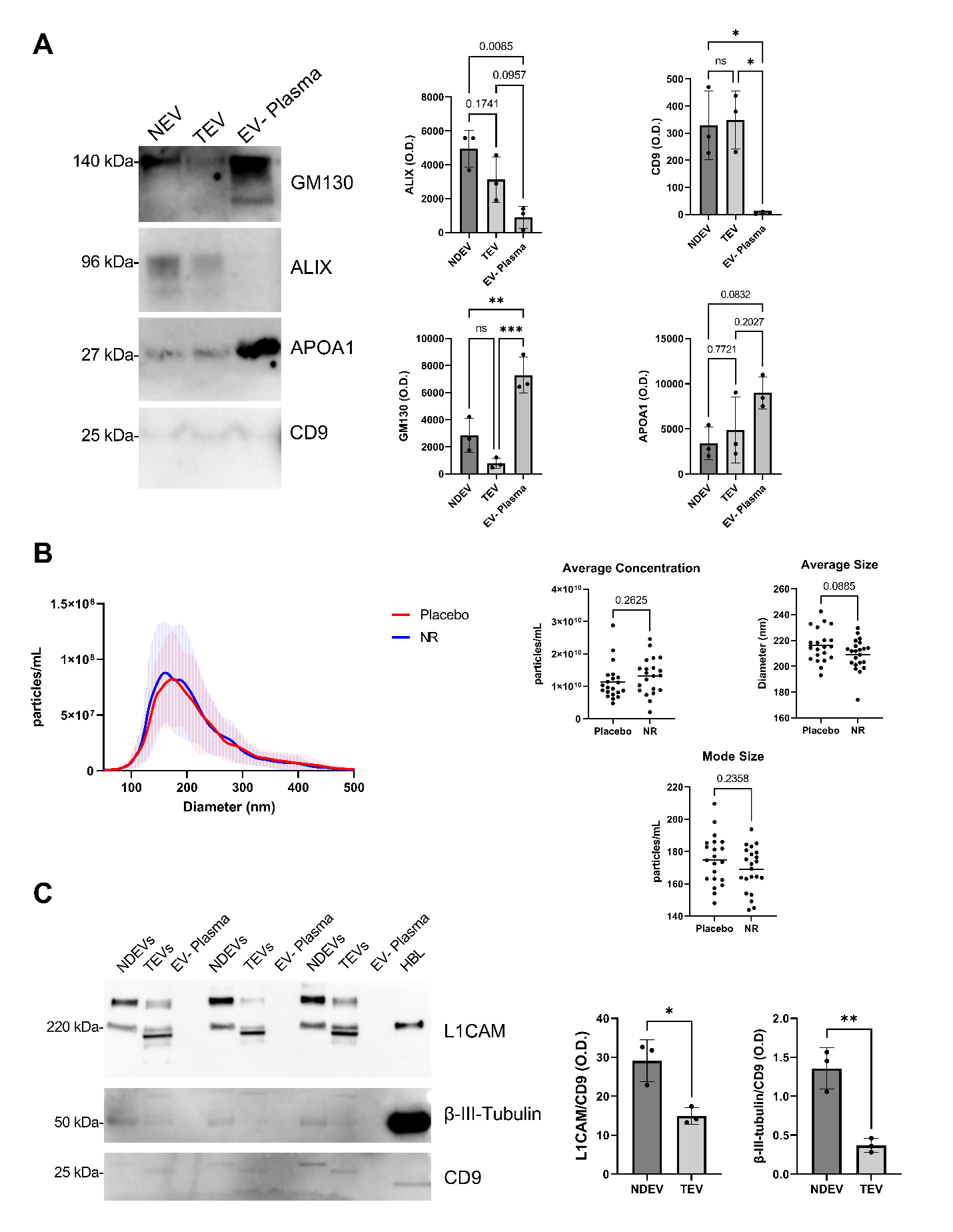


**Supplemental Figure 1:** Characterization of neuronal origin-derived extracellular vesicles (NEVs) isolated from plasma via L1CAM immunoprecipitation. (A) Representative western blot images and quantification of canonical positive (ALIX, CD9) and negative (GM130, APOA1) EV markers in NEVs, total EVs, and EV depleted plasma (ordinary one-way ANOVA, n=3). (B) Treatment group analysis of size distributions, mean size, mean concentration, and mode size of NEV isolates as determined by nanoparticle tracking analysis. Vertical lines depict SD. (C) Western blot images and quantification of neuronal marker β-III-tubulin, immunoprecipitation target L1CAM, and EV marker CD9 in NEVs, total EVs, and EV depleted plasma. Human brain lysate used as positive control for neuronal proteins (unpaired t-test, n=3). Images analyzed using FIJI/Image J. CD9 used as loading control; error bars depict SD; * indicates significance < 0.05; ** indicates significance < 0.005.

**Supplemental Figure 2:** Calibration curve used to interpolate NAD+ concentrations from Promega NAD+/NADH luciferase assay. NAD+ Standards were created at concentrations of 10 nM, 5 nM, 2.5 nM, 1.25 nM, and 0.625 nM.

**Supplemental Figure 3:** NADH levels in L1CAM+ EVs in responders only. Even when analyzing responders (defined as subjects with documented NAD+ increases in L1CAM+ EVs) only (n=9), levels of NADH in plasma extracellular vesicles enriched for neuronal origin remain relatively unchanged after oral NR supplementation when compared with the placebo condition (two-tailed paired t-test, p=0.44)
